# Long-term effectiveness of tumour necrosis factor-α inhibitor treatment for psoriatic arthritis in the UK: a multicentre retrospective study

**DOI:** 10.1093/rap/rky042

**Published:** 2018-10-17

**Authors:** Gavin Clunie, Iain B McInnes, Nick Barkham, Helena Marzo-Ortega, Yusuf Patel, Andrew Gough, Jon Packham, Stuart Kyle, Bruce Kirkham, Tom Sheeran, Helen Coope, Anna Bishop-Bailey, Neil McHugh

**Affiliations:** 1 Rheumatology, Addenbrookes Hospital, Cambridge; 2 Rheumatology, Glasgow Royal Infirmary, Glasgow; 3 Rheumatology, New Cross Hospital, Wolverhampton; 4 NIHR Leeds Biomedical Research Centre, Leeds Teaching Hospitals Trust Leeds, Leeds; 5 Leeds Institute of Rheumatic and Musculoskeletal Medicine, University of Leeds, Leeds; 6 Rheumatology, Hull Royal Infirmary, Hull; 7 Rheumatology, Harrogate District Hospital, Harrogate; 8 Rheumatology, Haywood Hospital, Stoke-on-Trent; 9 Rheumatology, North Devon Hospital, Barnstaple; 10 Rheumatology, Guy’s and St Thomas’ NHS Foundation Trust, London; 11 Rheumatology, Cannock Chase Hospital, Cannock; 12 Immunology & Dermatology, Novartis Pharmaceuticals UK Limited, Frimley; 13 Real World Evidence, pH Associates, Marlow; 14 Rheumatology, Royal National Hospital for Rheumatic Diseases, Bath, UK

**Keywords:** psoriatic arthritis, tumour necrosis factor inhibitors, observational study, swollen joints, tender joints, physician global assessment, patient global assessment, treatment persistence

## Abstract

**Objective:**

Real-world evidence of the long-term effectiveness of TNF-α inhibitor (TNFi) therapy in patients with PsA is limited. This study was conducted to describe patterns of TNFi therapy and treatment responses in patients with PsA treated in UK clinical practice.

**Methods:**

A multicentre, retrospective, observational cohort study of consenting patients treated with TNFi for PsA with ≥3 years follow-up from first TNFi initiation (observation period) was carried out in 11 UK National Health Service hospitals. Data were collected concerning baseline patient characteristics, PsA-related treatment pathways and TNFi treatment responses (PsA response criteria components: swollen/tender joint counts, physician and patient global assessments).

**Results:**

The mean age of patients (*n* = 141) was 50.3 (s.d.: 12.1) years (50% male). During a median observation period of 4.5 (range: 3.4–5.5) years, patients received a median of one (range: one to five) TNFi. Twelve-week response rates for first TNFi (where available) were as follows: 80% (*n* = 64/80) for swollen joint counts, 79% (*n* = 63/79) for tender joint counts, 79% (*n* = 37/47) for physician global assessments, 69% (*n* = 41/59) for patient global assessments and 79% (*n* = 37/47) for PsA response criteria. At the end of the observation period, the proportions of patients remaining on first, second, third and fourth/fifth TNFi were 56, 15, 5 and 3%, respectively; 21% of patients permanently discontinued TNFi therapy.

**Conclusion:**

Long-term TNFi therapy is generally well tolerated and may be effective; however, after initial TNFi failure, there appears to be progressively less benefit and more adverse effects with successive TNFi switches. Strategies are needed for effective therapy for PsA beyond the first TNFi failure.

**Key messages**

- Long-term treatment of PsA with TNF-α inhibitor may be effective in the majority of patients.
- Successive switching to an alternative TNF-α inhibitor therapy after initial TNF-α inhibitor failure provides progressively less benefit.
- New pathways for therapy in PsA need to be agreed beyond initial TNF-α inhibitor failure.

## Introduction

PsA is a progressive, chiefly autoinflammatory disease with variable clinical manifestations, including peripheral and axial joint inflammation, dactylitis, enthesitis, psoriasis and nail involvement [1]. The population prevalence of PsA has been estimated at between 0.05 and 0.25% in Europe and the USA [2]; PsA develops in up to 30% of patients with psoriasis, but the true prevalence of PsA-sine-psoriasis is unknown [3, 4]. A variety of treatment options are available for PsA, including NSAIDs, glucocorticoid injections and DMARDs, including conventional synthetic DMARDS (csDMARDs) and a rapidly changing landscape of targeted biologic DMARD and synthetic DMARD therapies [5–7]. TNF-α inhibitor (TNFi) therapy is an important treatment option for patients with PsA who are unresponsive to csDMARDs such as MTX. Randomized controlled trials have shown that TNFi therapies are more effective than placebo using a variety of assessment criteria [8–12], slowing disease progression and improving quality of life [5].

In the UK, the National Institute for Health and Care Excellence (NICE) recommends TNFi therapy for patients with active and progressive PsA who have peripheral arthritis with three or more tender joints and three or more swollen joints and have not responded to at least two csDMARDs, administered individually or in combination [3, 13]. NICE guidance recommends that TNFi therapy is discontinued in patients not achieving an adequate response using the PsA response criteria (PsARC) at 12 weeks, unless a psoriasis area and severity index 75 response has been achieved at 12 weeks [3, 13]. There is evidence from a number of national registries showing that many patients remain on the first TNFi over prolonged periods of time [14–20], although whether this reflects treatment effectiveness or the historical lack of alternative targeted therapies is unclear. Additional real-world evidence is required to understand the long-term effectiveness of TNFi therapy in routine clinical practice, particularly in patients who have been treated with multiple TNFi. A greater understanding of the longer-term outcomes of patients with PsA treated with TNFi will be useful for informing clinicians’ and patients’ decisions about treatment options after failure of TNFi, particularly given the recent availability of newer therapies targeted towards non-TNF inflammatory pathways [1, 21]. The aim of this study was to describe the patterns of first and subsequent TNFi use and response to long-term TNFi therapy in patients with PsA treated in routine clinical practice in the UK.

## Methods

### Study design and setting

A multicentre, retrospective, observational cohort study of patients treated with TNFi for PsA was carried out in 11 National Health Service (NHS) hospital Rheumatology departments in the UK, selected to include specialist and non-specialist centres with robust mechanisms of identifying suitable patients with comprehensive medical records, and providing a wide geographical distribution to ensure generalizability of results. The study included eligible patients with PsA with long-term (≥3 years) follow-up data available after initiation of the first TNFi. Data collection took place between 24 April 2015 and 1 December 2015.

### Patients

Patients who had been diagnosed with PsA according to ClASsification criteria for Psoriatic ARthritis (CASPAR) criteria [22], who were aged ≥18 years at the start of TNFi treatment for PsA and first treated with a TNFi between 1 January 2010 and 31 December 2011 (to focus on more recent rather than historic clinical practice while ensuring a minimum of 3 years of follow-up) were eligible for inclusion in the study. Consecutive (by date) eligible patients were identified from hospital medical records by the direct clinical care team and approached to provide consent to participate in the study, with a maximum of 20 patients recruited per centre to take into account the different patient population sizes at each centre while maintaining a representative geographical distribution of study patients. Patients gave written informed consent in accordance with the Declaration of Helsinki (updated 2008) for retrospective data collection from medical records according to a protocol approved by the Research Ethics Committee (REC) East of England – Norfolk (REC reference number 15/EE/0029).

#### Sample size

Owing to limited availability of studies reporting longitudinal data for long-term outcomes of TNFi therapy according to PsARC response and the single-cohort study design, the target sample size of 150 patients was selected on a pragmatic basis using an anticipated PsARC response rate at 12 weeks after initiation of first TNFi of 80% (estimated 95% CIs of 73–87%) based on the results of previous studies [8, 10].

### Data collection

Data describing baseline patient demographic and clinical characteristics, PsARC response components and PsA-related treatment pathways after initiation of first TNFi therapy were sourced from medical records, including all relevant paper notes and electronic databases as appropriate in each participating hospital. All data were recorded in anonymized-coded form on standard data collection forms designed for the study. Patient demographic and clinical characteristics at baseline (data recorded at the time of initiation or closest before TNFi initiation) included: age at diagnosis and TNFi initiation, gender, smoking history, body mass index, co-morbidities and PsA-related clinical manifestations. The PsARC response components recorded at the baseline PsA assessment visit (defined as the Rheumatology outpatient visit recorded at the time of initiation or closest before TNFi initiation, during which one or more PsARC components were recorded) included: swollen joint count (SJC), tender joint count (TJC), physician global assessment (PGA) score and patient global assessment (PtGA) score. Data on treatment pathways during the observation period (from initiation of first TNFi until data collection) included: TNFi therapy, duration of treatment for each different TNFi received, reasons for discontinuing TNFi therapies, concomitant medication, PsARC response components, concomitant csDMARD therapy and PsA therapies after TNFi discontinuation.

### Response to TNFi therapy

#### PsARC response

A PsARC response is achieved if no component is worse and at least two of the following apply: improvement of ≥30% in TJC or SJC (at least one required, based on 68/66 joint count) and/or improvement in PGA and/or PtGA of at least one point on a five-point Likert scale [10]. The TJC and SJC were assessed using different scores (78/76, 68/66 and 28/28 joint counts) at different centres and by different clinicians. For the purposes of assessing response to treatment, the 68/66 and 78/76 joint counts were considered to be equivalent, as previously described [23] and pooled for analysis, whereas 28/28 joint counts were analysed separately (see supplementary methods, available at *Rheumatology Advances in Practice* online). The PGA and PtGA scores were evaluated using different scales [five-point Likert scale, 10-point visual analog scale (VAS) or 100-point VAS] at different centres and by different clinicians. Given that each scale is linear, thresholds for improvement or worsening of the PGA and PtGA scores of at least two points on a 10-point VAS and ≥20 points on a 100-point VAS were considered to be equivalent to least one point on a five-point Likert scale (only when assessed using the same scale at baseline and post-TNFi assessment visits). The PsARC responses after initiation of first TNFi were evaluated based on the percentage increase or decrease in the number of SJC and TJC and the number of points increase or decrease in PGA and PtGA, comparing the scores for the post-TNFi initiation time points with baseline scores (see supplementary methods, available at *Rheumatology Advances in Practice* online).

#### Treatment persistence

For assessment of the duration of therapy for each TNFi, any temporary breaks in TNFi therapy were disregarded and the duration of therapy for a particular TNFi was taken as the time from initiation until the date of formal discontinuation or the end of the observation period (whichever was soonest).

### Statistical analyses

All analyses were descriptive in nature and performed on the available data, with no imputation of missing values; the denominators for all analyses where data were missing are presented in the relevant figures, tables or text. Quantitative variables are presented as the median [interquartile range (IQR) or range] or arithmetic mean (s.d.). Nominal variables are presented as the frequency (percentage) and ordinal variables as the median (IQR).

## Results

### Baseline patient demographic and clinical characteristics

One hundred and forty-one patients from 11 hospitals in the UK treated with first TNFi between January 2010 and December 2011 were included in the study. The baseline patient demographic and clinical characteristics at initiation of first TNFi therapy are presented in Table 1. The mean age of patients at initiation of first TNFi was 50.3 (s.d.: 12.1) years; 50% of patients were male, 10% of patients were current smokers, and median disease duration was 5.7 (IQR: 2.0–11.6) years. At initiation of first TNFi, 53% of patients had at least one recorded co-morbidity, and 92% of patients had at least one PsA-related clinical manifestation (psoriasis, peripheral arthritis, nail involvement, enthesitis, dactylitis, axial arthritis). At least one baseline PsARC response component was recorded before initiation of first TNFi for 134 (95%) patients, and all four response components were recorded for 87 (62%) patients (see supplementary Fig. S1, available at *Rheumatology Advances in Practice* online), with a mean time from baseline PsA assessment to initiation of first TNFi of 6.2 (s.d.: 5.2) weeks (*n* = 134).

**Table 1:** Baseline patient demographic and clinical characteristics at initiation of first TNF-α inhibitor

Characteristic	Overall patient population (*n* = 141)
Age, mean (s.d.), years	50.3 (12.1)
Males, *n* (%)	70 (50)
Smokers, *n* (%)	
Current	14 (10)
Former	34 (24)
Never	60 (43)
Unknown	33 (23)
BMI, mean (s.d.), kg/m^2^	28.7 (5.4) (*n*=68)
PsA disease duration, median (IQR), years	5.7 (2.0–11.6) (*n*=138)
Co-morbidities, *n* (%)	
Hypertension	39 (28)
Obesity	30 (21)
Hypercholesterolaemia	24 (17)
Diabetes/high blood glucose	17 (12)
Depression	13 (9)
Coronary heart disease	8 (6)
None	66 (47)
PsA-related clinical manifestations, *n* (%)	
Psoriasis	118 (84)
Peripheral arthritis	105 (74)
Nail involvement	42 (30)
Enthesitis	27 (19)
Dactylitis	26 (18)
Axial arthritis	17 (12)
None recorded	11 (8)
Swollen joint count, median (IQR)	(*n*=128)
68/66 or 78/76	7.5 (5.0–12.0)
28/28	6.5 (3.8–8.5)
Tender joint count, median (IQR)	(*n*=128)
68/66 or 78/76	17.5 (8.0–27.3)
28/28	15.5 (10.0–22.3)
Physician global assessment score, median (IQR)	(*n*=90)
5-point Likert scale	4 (3–4) (*n*=77)
100-point VAS	39.5 (28.5–53.5) (*n*=6)
10-point VAS	3 (3.0–3.5) (*n*=7)
Patient global assessment score, median (IQR)	(*n*=113)
5-point Likert scale	4 (3–4) (*n*=79)
100-point VAS	65.0 (42.5–75.0) (*n*=34)

Data are presented as *n* (%) unless stated otherwise. IQR: interquartile range; VAS: visual analog scale.

**Fig. 1:**
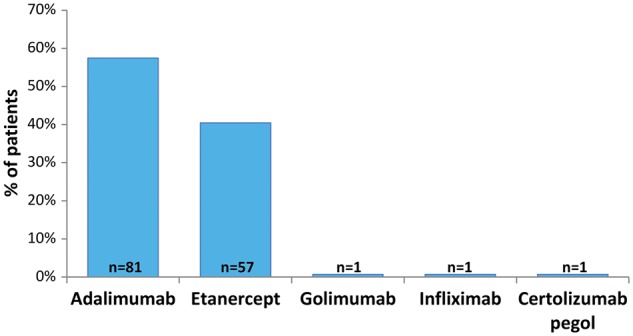
First TNF-α inhibitor therapy Patients were first treated with a TNFi between 1 January 2010 and 31 December 2011. TNFi: TNF inhibitor.

Most patients (76/141) were observed for between 4 and 5 years after initiation of the first TNFi, with a median observation period of 4.5 (range: 3.4–5.5) years. The most common first TNFi therapies were adalimumab (*n* = 81) and etanercept (*n* = 57), as shown in Fig. 1 (no patients were treated with TNFi biosimilars). Patients received a median of one (range: one to five) different TNFi during the observation period. The majority of patients (67%) received one TNFi, with 11% of patients receiving three or more TNFi therapies during the observation period (supplementary Table S1, available at *Rheumatology Advances in Practice* online).

### Response to TNFi treatment

Response to TNFi treatment was evaluated based on the available PsARC response components.

#### PsARC component responses

**Fig. 2:**
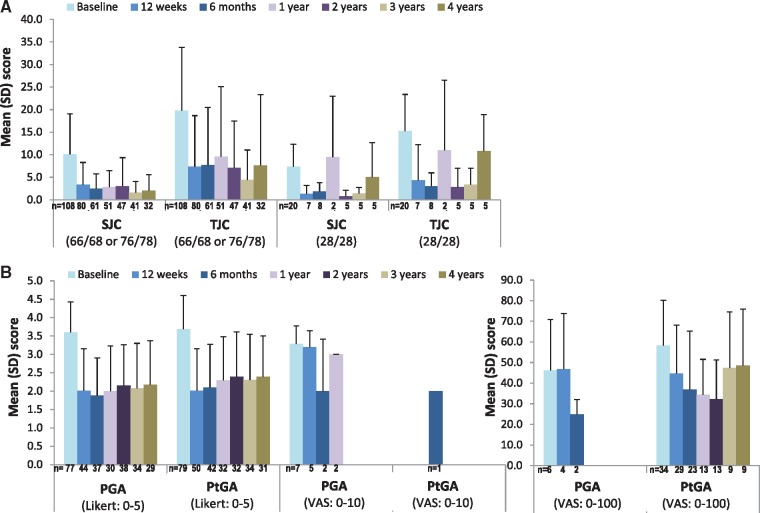
Scores for individual PsA response criteria components during the study observation period (**A**) Swollen and tender joint counts. (**B**) Physician and patient global assessments. PGA: physician global assessments; PsARC: PsA response criteria; PtGA: patient global assessments; SJC: swollen joint counts; TJC: tender joint counts.

**Fig. 3:**
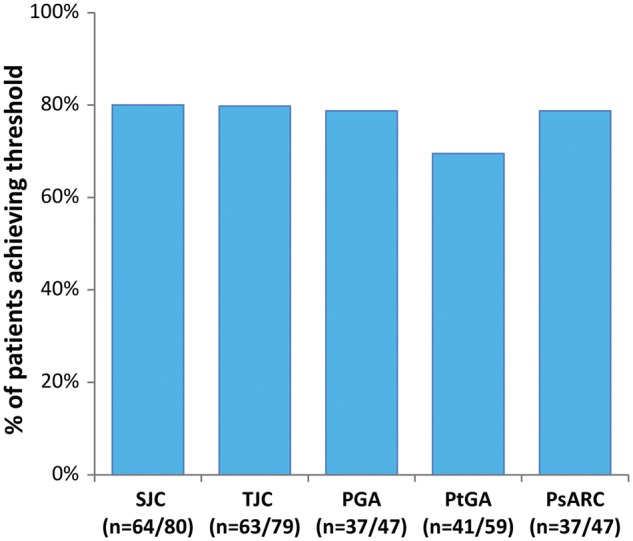
Response to first TNF-α inhibitor at 12 weeks Bars represent the proportions of patients achieving the following thresholds: joint count ≥30% improvement; global assessment improvement of at least one point on five-point Likert scale, ≥2 points on 10-point VAS, ≥20 points on 100-point VAS. PGA: physician global assessments; PsARC: PsA response criteria; PtGA: patient global assessments; SJC: swollen joint counts; TJC: tender joint counts; TNFi: TNF inhibitor; VAS: visual analog scale.

Reflecting the real-world design of this study, the PsARC components were not recorded for all patients at all time points (see Fig. 2). The PsARC responses could be determined only at 12 (s.d.: 4) weeks in 47 patients and at 1 year (s.d. 8 weeks) in 27 patients (see supplementary Fig. S1, available at *Rheumatology Advances in Practice* online). The 12-week response rates for first TNFi were 80 and 79%, respectively, for SJC and TJC; 79 and 69%, respectively, for PGA and PtGA; and 79% for PsARC response, as shown in Fig. 3. Similar response rates were observed at all time points evaluated during the observation period (see supplementary Fig. S2, available at *Rheumatology Advances in Practice* online).

#### TNFi treatment persistence

**Fig. 4:**
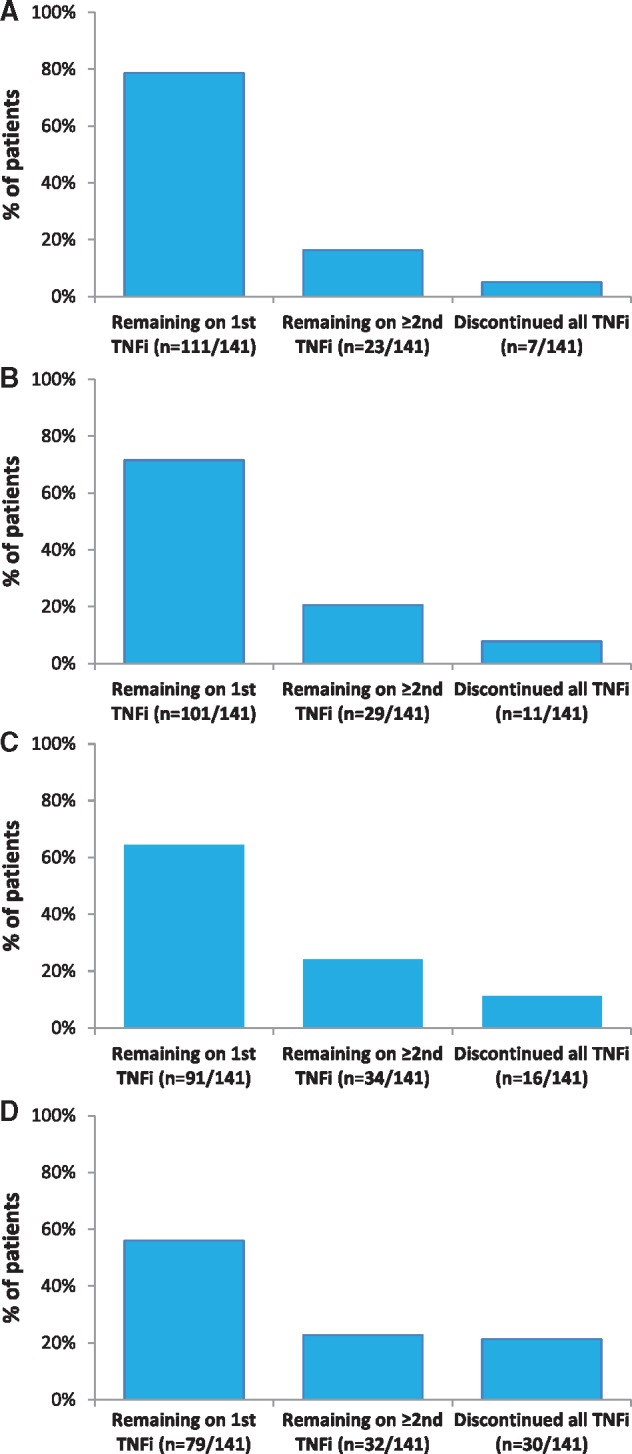
TNF-α inhibitor treatment persistence after initiation of first TNF-α inhibitor (**A**) Percentage of patients remaining on TNFi treatment at 1 year. (**B**) Percentage of patients remaining on TNFi treatment at 2 years. (**C**) Percentage of patients remaining on TNFi treatment at 3 years. (**D**) Percentage of patients remaining on TNFi treatment at the end of the observation period. TNFi: TNF inhibitor.

The proportions of patients who remained on the first TNFi at 1, 2 and 3 years post-initiation were 79, 72 and 65%, respectively, as shown in Fig. 4. At the end of the observation period, 56% of patients remained on the first TNFi, 15% of patients were on the second TNFi, 5% of patients were on the third TNFi, 3% of patients were on at least the fourth different TNFi (see Fig. 4), and 21% (30/141) of patients had permanently discontinued TNFi therapy [8% (*n* = 11) after the first, 9% (*n* = 13) after the second, 3% (*n* = 4) after the third and 1% (*n* = 2) after the fourth TNFi].

The mean duration of TNFi therapy was 53.6 (s.d.: 6.6) months in patients remaining on the first TNFi (*n* = 79) and 19.2 (s.d.: 16.6) months in patients who discontinued the first TNFi [*n* = 62; six patients (9.7%) had discontinued by 3 months]. The most common reasons for discontinuing the first TNFi were lack of/loss of efficacy (45%) and adverse events (18%), as shown in Table 2. The mean duration of second TNFi therapy was 31.6 (s.d.: 16.6) months in patients remaining on the second TNFi (*n* = 21) and 11.6 (s.d.: 13.1) months in patients who discontinued the second TNFi [*n* = 24; five patients (21%) had discontinued by 3 months]. The most common reasons for discontinuation of the second TNFi were lack of/loss of efficacy (44%) and adverse events (40%; see Table 2). The mean duration of third TNFi therapy was 18.1 (s.d.: 15.2) months in patients remaining on the third TNFi (*n* = 7) and 7.8 (s.d.: 7.6) months in patients who discontinued the third TNFi [*n* = 9; four patients (44%) had discontinued by 3 months]. The most common reason for discontinuation of the third TNFi was adverse events (56%; see Table 2). The mean duration of fourth/fifth TNFi therapy was 23.4 (s.d.: 7.6) months in patients remaining on the fourth/fifth TNFi (*n* = 4) and 8.8 (s.d.: 9.6) months in patients who discontinued the fourth/fifth TNFi [*n* = 3; one patient (33%) had discontinued by 3 months]. The most common reason for discontinuation of the fourth/fifth TNFi was adverse events (67%; see Table 2).

**Table 2:** Reasons for discontinuing TNF-α inhibitor therapy

Reason for discontinuation^a^	TNFi therapy			
	First TNFi (*n* = 62)	Second TNFi (*n* = 25)	Third TNFi (*n* = 9)	Fourth/fifth TNFi (*n* = 3)
Lack of/loss of efficacy	28 (45)	11 (44)	3 (33)	1 (33)
Adverse events	11 (18)	10 (40)	5 (56)	2 (67)
Injection site reactions	6 (10)	1 (4)	–	–
Infection/recurrent infection	7 (11)	2 (8)	–	–
Other	13 (21)	3 (12)	3 (33)	–
Not recorded	1 (2)	–	–	–

Data are presented as (%); ^a^not mutually exclusive. TNFi: TNF-α inhibitor.

### Co-prescription of csDMARDs

DMARDs were co-prescribed with the first TNFi in 102/137 (74%) patients (77 patients received MTX; see supplementary Table S2, available at *Rheumatology* online). The timing of introduction of MTX relative to TNFi initiation could be assessed in 74 patients; of these, 64/74 (86%) patients were already treated with MTX at first TNFi initiation, 5/74 (7%) patients started MTX at the same time as the first TNFi, and 5/74 (7%) patients started MTX after initiation of the first TNFi.

### Treatments for PsA after discontinuation of TNFi

Of the 30 patients (21%) who discontinued TNFi therapy during the observation period, 7/30 (23%) had received no further treatments for PsA, 1/30 (3%) patients received NSAIDs only, 12/30 (40%) received csDMARDs only, 1/30 (3%) received a synthetic therapies DMARD only, 3/30 (10%) received another biologic DMARD only, and 6/30 (20%) patients received csDMARDs in addition to one or more other treatments for PsA (see supplementary Table S3, available at *Rheumatology* online).

## Discussion

The baseline demographics and clinical characteristics of patients with PsA initiated on first- TNFi were broadly similar to previously published observational studies in PsA populations [15–20, 24–29]. Although PsARC is the instrument recommended by NICE for assessing response to TNFi therapy in patients with PsA [3] and was developed to monitor response to treatment in clinical trials [10, 30], only one previous longitudinal observational study has reported PsARC responses to TNFi therapy [26]. Despite NICE recommendations that TNFi therapy is discontinued at 12 weeks in patients not achieving an adequate response using PsARC, sufficient data to assess PsARC were available in only 33% of patients at 12 weeks and in only 19% of patients at 1 year, despite nearly 80% of patients remaining on the first TNFi at 1 year. These findings suggest that NICE guidance is not widely followed when assessing response to treatment in patients with PsA in routine UK clinical practice. However, in the small proportion of patients with PsARC assessments available, the response rates were ≥75% at all time points evaluated. Given the extent of missing data, caution is warranted in interpreting these results; however, the similarity between the proportions of patients achieving improvements in individual PsARC components and the proportion of PsARC responders might suggest that reporting bias was unlikely. Furthermore, the response rates we observed are consistent with the reported PsARC response rates of ≥60% reported in clinical trials for time periods of ≤24 weeks [8–12, 31]. The response rates are also consistent with the 56–64% PsARC response rates to different TNFi therapies reported in a prospective observational open-label study of patients with PsA remaining on treatment after 5 years [26]. Other previous observational studies have assessed short-term responses to TNFi treatment using a variety of clinical measures of disease activity, including ACR20/50/70, EULAR and DAS28 [15, 16, 18, 25, 26, 28, 32]. Owing to the differences in the response components (in particular, use of the 28 joint count for DAS28, which excludes joints commonly affected in PsA, such as hips, feet and DIP joints) and classification of overall response for these different measures [33], it is difficult to compare previous response rates directly with our study.

Identification of the underlying reasons for the incomplete reporting of PsARC components was beyond the scope of the present study; however, variability in documenting PsARC component assessments most probably reflects a combination of real-world factors. These include the complex clinical manifestations of PsA; the lack of PsA guidelines and standards and lack of consensus on how and when to measure disease activity and treatment response; and a variety of patient-related and service-related factors (including constraints on outpatient clinic capacity, impacting on appointment durations and staff expertise). However, this has important implications for patients, because poor responses should be documented clearly in order to support treatment decision-making.

The EULAR recommendations for the treatment of PsA highlight the importance of regular (between 1- and 3-monthly) monitoring of disease activity and appropriate adjustment of therapy [6]. EULAR also recommends the use of composite measures including joint counts for monitoring disease activity, while acknowledging a lack of consensus on the best way to monitor disease activity across different tissues [6]. Joint counts are an important component of many composite PsA response measures [33]; consistent with this, we found that joint counts were more frequently recorded than PtGA and PGA at all time points evaluated. However, it has previously been shown that joint counts are relatively poor at predicting changes in treatment [34], reflecting the fact that peripheral arthritis is only one of the symptoms of PsA [7, 35]. Consistent with this, most patients included in this study had documented psoriasis and peripheral arthritis, and ∼20% of patients had documented enthesitis and dactylitis. Therefore, although the joint count responses in our study were broadly consistent with the available PsARC response rates, caution is warranted in interpretation of these data as a measure of treatment effectiveness given the poor sensitivity of joint counts in predicting treatment changes. Furthermore, it seems likely that the variability in recording PsARC components, at least in part, reflects the ongoing lack of consensus regarding the use of composite compared with unidimensional scoring systems focused on the specific disease characteristics and health-related quality of life of individual patients [6, 36]. The limited documentation of PsARC might suggest poor broad applicability in routine clinical practice and may call into question its suitability as the recommended instrument for assessing response to treatment in NICE PsA guidance. Patient-reported outcome measures such, as the routine assessment of patient index data 3, might be more suitable for assessing patients in routine clinical practice [37, 38].

Most patients received co-prescribed MTX during treatment with the first TNFi, and the timing of therapy suggests that MTX was maintained as supportive therapy rather than being prescribed as a rescue therapy or to improve persistence in most of these patients. Although co-prescription of MTX with TNFi for the treatment of PsA is reported to be common, it is notable that evidence from clinical trials and observational studies suggests that co-prescription of MTX does not improve the response to TNFi therapy [39].

The majority of observational studies have reported treatment persistence as a surrogate for clinical effectiveness in assessing longer-term responses to TNFi treatment; most studies demonstrated that ≥50% of patients remained on TNFi therapy a minimum of 2 years after initiation of first TNFi [14–19, 27, 28, 32, 40], with only two studies reporting median treatment persistence of <2 years [20, 29]. One study reported that 86.7% of patients treated with etanercept who had achieved remission by month 6 (defined as zero joints with synovitis) remained in remission (discontinuation for any cause was defined as non-response) at month 66 of follow-up [40]. This suggests that once remission is achieved with TNFi therapy it is maintained in the longer term; however, the study was originally designed to assess treatment adherence with defined criteria for study withdrawal [40], suggesting that the results might not be reflective of routine clinical practice. Consistent with the majority of studies, we found that ≥75% of the patients remained on TNFi therapy after a median of 4.5 years follow-up (∼50% of all patients remained on the first TNFi). Furthermore, the overall proportion of patients remaining on treatment was similar to the response rates for the different PsARC components, which may support treatment persistence as a marker for treatment effectiveness in our study. However, it cannot be excluded that treatment persistence may also reflect a placebo effect for some patients or a lack of alternative therapies targeting different inflammatory pathways during the observation period of this long-term study. In this context, it is notable that the average TNFi treatment duration in patients switching TNFi therapy decreased and the proportion of patients discontinuing TNFi therapy within 3 months of initiation increased with subsequent lines of TNFi, with lack of/loss of efficacy and intolerance being the most commonly recorded reasons for discontinuation across successive TNFi therapies. This observation is consistent with previous registry data [15, 20, 29, 41], suggesting many patients are more resistant and/or intolerant to multiple TNFi after initial treatment failure. Taken together with the results of previous studies, our results suggest that long-term TNFi therapy is well tolerated and may be effective in ≥50% of patients. However, although switching to a second TNFi after failure on first therapy owing to either lack of efficacy or intolerance is recommended by the British Society of Rheumatology/British Health Professionals in Rheumatology guidelines [42] and EULAR guidelines [6], our study adds to the available evidence suggesting that switching TNFi therapy after failure on first TNFi may not provide prolonged benefits for many patients.

At the end of the observation period, approximately one-fifth of patients had permanently discontinued TNFi therapy, with the majority of these going on to receive only conventional DMARD therapy, emphasizing the lack of alternative targeted therapies for PsA during the observation period of the present study. The significant minority of patients who permanently discontinued TNFi and the number of patients treated with more than two lines of TNFi suggest a considerable unmet clinical need for the treatment of PsA during the observation period of the present study. However, the recent availability of newer and emerging therapies targeting different inflammatory pathways, including ustekinumab (IL-12/IL-23 inhibitor), secukinumab (IL-17A inhibitor) and apremilast (phosphodiesterase inhibitor), will provide greater opportunities for more individualized treatment in the future [1, 21].

### Limitations of the study

Given that informed patient consent was required for this study, selection bias was possible, which may have influenced the study end points. All data were sourced retrospectively from patient medical records, resulting in varying degrees of missing data for different variables, a recognized limitation of retrospective epidemiological studies. The PsARC response was assessed only if the required variables were recorded within the permitted time windows for each time point and therefore data were not available for all patients, which might have biased response rates. Although most patients had tender/swollen joint counts assessed using the 68/66 or the 78/76 joint counts (considered to be equivalent to the 68/66 joint count for the purposes of the present study), a small proportion of patients had joint counts assessed using the 28/28 joint count, which may be unreliable owing to the likelihood of missing tender and swollen joints at baseline and/or follow-up, particularly given the significant involvement of feet in PsA [35]. Furthermore, we cannot exclude the possibility that the documentation of PsARC response components may have differed in patients responding and not responding to TNFi therapy. Temporary breaks in TNFi therapy were disregarded when calculating the duration of treatment for each TNFi, and the number and duration of temporary breaks is unknown. Although the impact is likely to be small, this will have resulted in an overestimation of overall TNFi treatment persistence. Treatment persistence might not reflect a favourable effect of TNFi on PsA disease severity, because patients might have remained on treatment owing to the lack of alternative non-TNFi biologic therapies during the observation period, and we were unable to correlate treatment persistence with PsARC responses owing to the limited data available. The patients in our cohort commenced first TNFi between 2010 and 2011 to ensure a minimum of 3 years of follow-up; as a result, our data may not be reflective of current clinical practice.

### Conclusion

Long-term TNFi therapy is well tolerated and may be effective in ≥50% of patients based on TNFi persistence. However, changing to an alternative TNFi therapy may provide less benefit and may result in more adverse effects for some patients after failure of the first and subsequent TNFi. The recent availability of newer and emerging therapies targeting different inflammatory pathways will enable more individualized treatment for patients with PsA in the future. Our data also suggest that NICE recommendations regarding the assessment of response to TNFi therapy based on PsARC response are not widely followed, which might reflect poor broad applicability in the routine clinical practice setting. Given the changing landscape of PsA management in the UK, a future study evaluating the impact of these newer targeted therapies on outcomes in patients with PsA not adequately controlled by conventional DMARDs and for whom TNFi therapy has not been successful is warranted.

## Supplementary Material

Supplementary Data
