# Supplementary material for: Long-term effectiveness of tumour necrosis factor-α inhibitor treatment for psoriatic arthritis in the UK: a multicentre retrospective study
Source: Rheumatol Adv Pract. 2018 Oct 17;2(2):rky042. doi: 10.1093/rap/rky042 (PMC6649900; doi:10.1093/rap/rky042)
Supplement: Supplementary Data [file rky042_supp.docx]

**SUPPLEMENTARY DATA**

**Supplementary Methods**

***PsARC response***

A PsARC response is achieved if no component is worse and at least 2 of the following apply: improvement of ≥30% in TJC or SJC (at least one required) and/or an improvement in PGA and/or PtGA of at least 1 point on a 5-point Likert scale [1]. SJC and TJC were assessed using different scores (76/78, 66/68 and 28/28 joint counts) at different centres and by different clinicians. For the purposes of assessing treatment response, the 66/68 joint count was considered to be equivalent to the 76/78 joint count since the only difference between them is inclusion of both toe joints in the 76/78 count (interphalangeal and distal interphalangeal joints).The data for the 66/68 and 76/78 joint counts were pooled for analysis and 28/28 joint counts were analysed separately. This approach is supported by results of a retrospective audit in which PsARC response rates were identical when evaluated using either the 76/78 or 66/68 joint counts[2]. PGA and PtGA scores were evaluated using different scales (5-point Likert scale, 10-point visual analogue scale [VAS] or 100-point VAS) at different centres and by different clinicians. Since each scale is linear, for the purposes of evaluating PsARC responses, a threshold for improvement or worsening of ≥1 point in the PGA and PtGA scores using a 5-point Likert scale was considered to be equivalent to ≥2 points when using a 10-point VAS and ≥20 points when using a 100-point VAS (only where patients were assessed using the same scale at baseline and post-TNFi assessment visits; patients assessed using different scales at different time points were excluded from the relevant analyses). PsARC responses following initiation of first TNFi were evaluated based on the percentage increase or decrease in the number of SJC and TJC and the number of points increase or decrease in PGA and PtGA comparing the scores for the post-TNFi initiation time-points with baseline scores. The variables used to define PsARC responses during the observation period following initiation of first TNFi were captured for the assessments recorded closest to the following response time points with pre-defined time windows: response at 12 weeks (12 weeks +/- 4 weeks); response at 6 months (6 months +/- 8 weeks); response at 12 months (12 months +/- 8 weeks); response at 2 years (2 years +/- 8 weeks); response at 3 years (3 years +/- 8 weeks); response at 4 years (4 years +/- 8 weeks).

**Supplementary Tables**

**Supplementary table S1. Number of TNFi received during observation period**

| **Different TNFi received** | **Number of patients** | **% (n=141)** |
| --- | --- | --- |
| **1** | 95 | 67.4% |
| **2** | 30 | 21.3% |
| **3** | 10 | 7.1% |
| **4 or 5** | 6 | 4.2% |
| **Total** | 141 | 100.0% |

TNFi: tumour necrosis factor inhibitor

**Supplementary table S2. csDMARDs co-prescribed with first TNFi therapy**

| **csDMARD** | **Number of patients taking csDMARDS^a^** | **% (n=102)*** |
| --- | --- | --- |
| Methotrexate | 77 | 75% |
| Sulfasalazine | 23 | 23% |
| Leflunomide | 25 | 25% |
| Hydroxychloroquine | 5 | 5% |

^a^Not mutually exclusive. TNFi: tumour necrosis factor inhibitor; csDMARD: conventional synthetic disease-modifying antirheumatic drug

**Supplementary table S3. Treatments for PsA after discontinuation of TNFi**

| **PsA treatments** | **Number of patients** | **% (n=30)^c^** |
| --- | --- | --- |
| csDMARDS only | 12 | 40% |
| csDMARD + NSAIDs^a^ | 4 | 13% |
| csDMARD + glucocorticoid + other bDMARD^a^ | 2 | 7% |
| tsDMARD only^b^ | 1 | 3% |
| NSAIDs only | 1 | 3% |
| Other bDMARD only | 3 | 10% |
| None | 7 | 23% |
| **Total** | 30 |  |

^a^Unknown if concurrent or sequential. ^b^Received as part of a clinical trial. ^c^Does not sum to 100% due to rounding. TNFi: tumour necrosis factor inhibitor; bDMARD: biologic DMARD; tsDMARD: targeted synthetic DMARD; other bDMARDS include: rituximab, ustekinumab, brodalimumab, secukinumab, abatacept.

**Supplementary Figure S1. Summary of the patients included in analyses of baseline data and PsARC component responses following TNFi initiation**. **
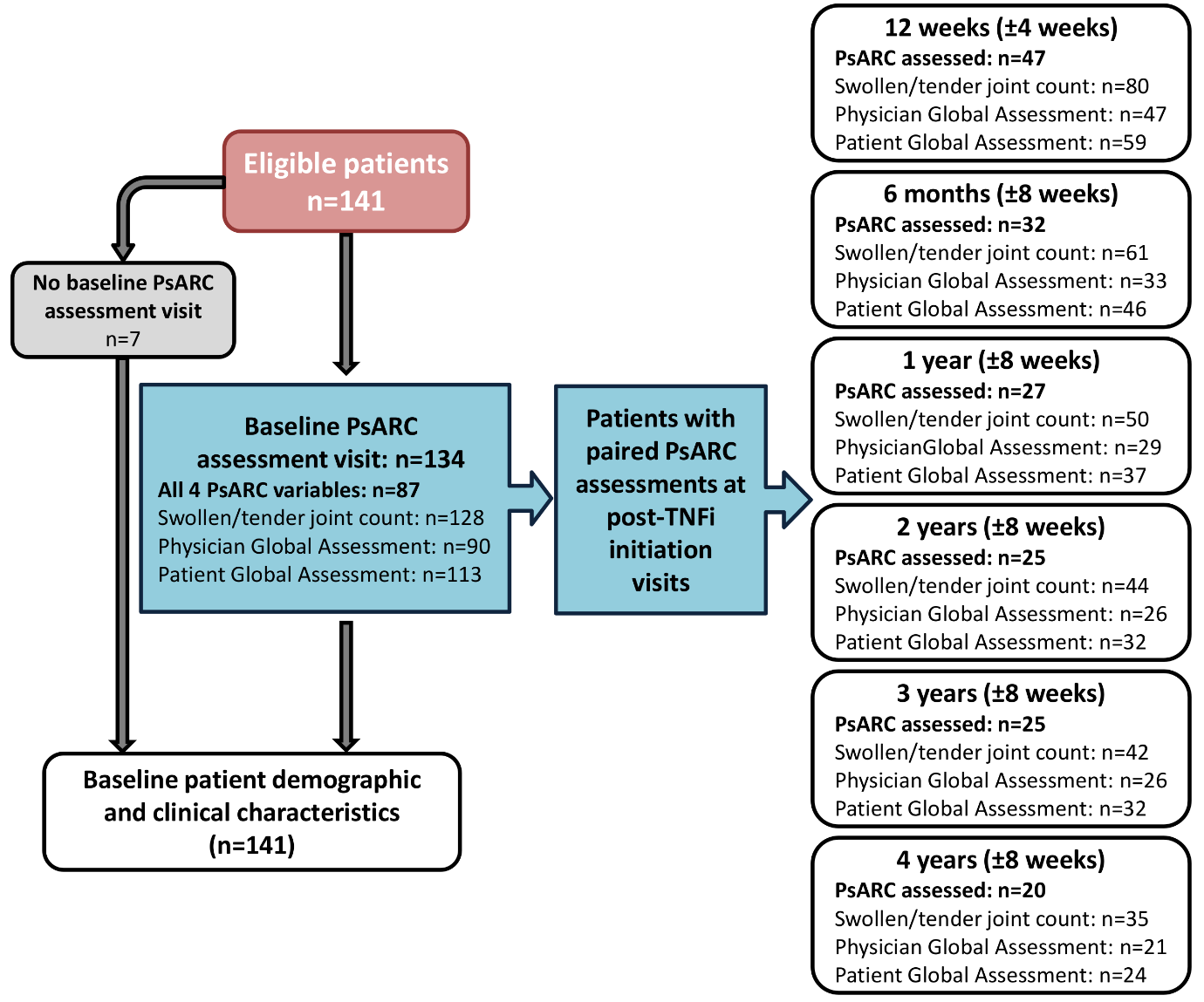
**

Of the 128 patients with baseline swollen/tender joint counts, 20 (15.6%) had been assessed using the 28/28 joint count. TNFi: tumour necrosis factor inhibitor; PsARC: PsA response criteria

**Supplementary Figure S2 PsARC component responses during the observation period**.


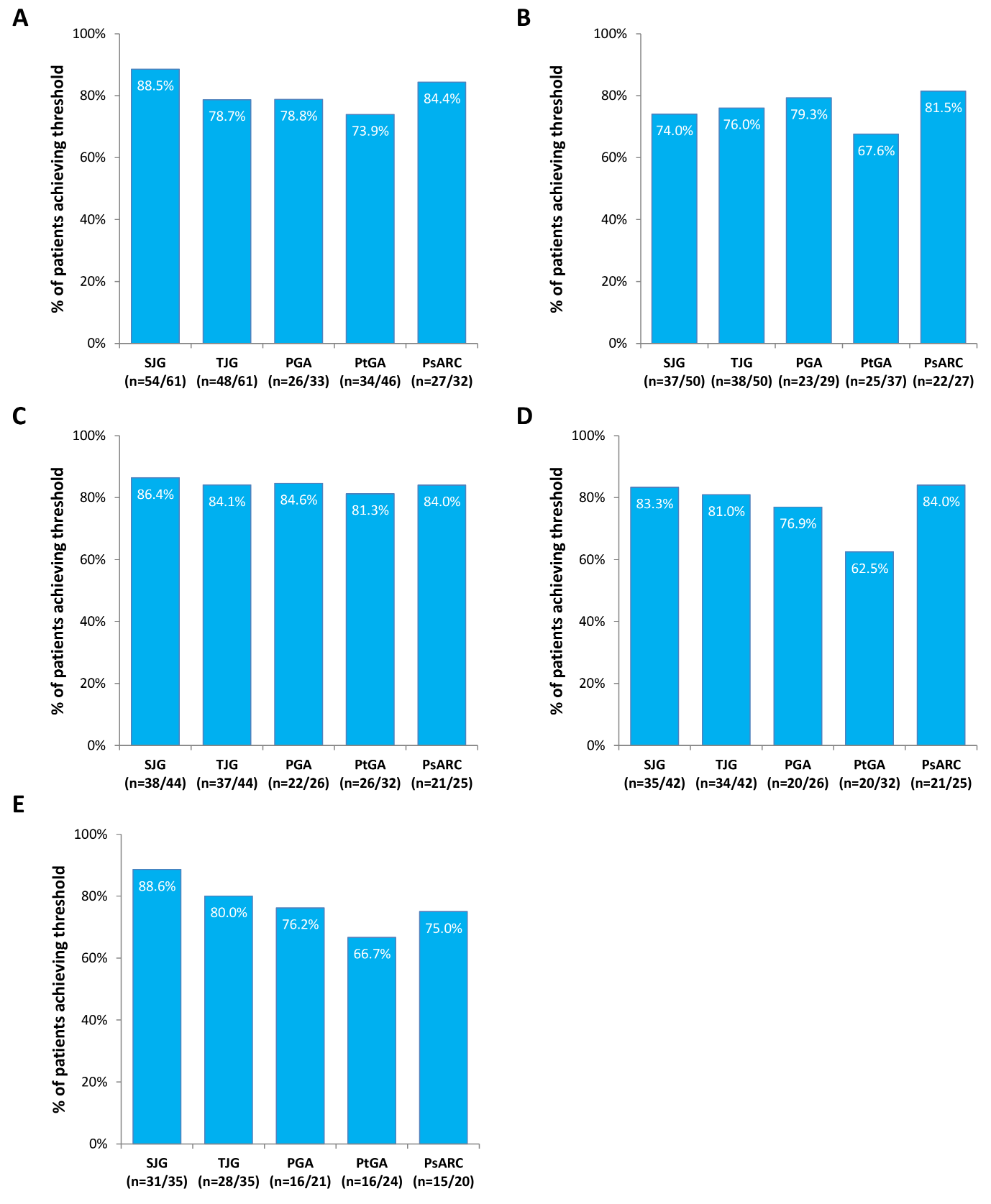


A: 6-month responses; B: 12-month responses; C: 2-year responses; D: 3-year responses; E: 4-year responses. Bars represent the proportions of patients achieving responses at different time points compared with baseline, based on the following thresholds: joint count ≥30% improvement; Global Assessment improvement of ≥1 point on 5-point Likert, ≥2 points on 10-point VAS, ≥20 points on 100-point VAS. VAS: visual analogue scale; SJC: swollen joint count; TJC: tender joint count; PGA: Physician Global Assessment; PtGA: Patient Global Assessment; PsARC: PsA response criteria.

**References**

1. Mease PJ, Goffe BS, Metz J et al. Etanercept in the treatment of psoriatic arthritis and psoriasis: a randomised trial. Lancet 2000;356:385–90.

2. Day SH, Butt S, Deighton C, Gadsby K. The Consequence of Using Different Methods of Joint Assessment on the Eligibility for Access to Anti-TNF in Psoriatic Arthritis [Internet]. In: Rheumatology. 2010 [cited 2016 Mar 11]. page i17.Available from: https://rheumatology.oxfordjournals.org/content/49/suppl_1/i14.full
